# Calibration of Inverted Asphalt Pavement Rut Prediction Model, Based on Full-Scale Accelerated Pavement Testing

**DOI:** 10.3390/ma16020814

**Published:** 2023-01-13

**Authors:** Zhenqiang Han, Aimin Sha, Liqun Hu, Wei Jiang

**Affiliations:** 1 School of Highway, Chang’an University, Xi’an 710064, China; 2 Key Laboratory for Special Area Highway Engineering of Ministry of Education, Chang’an University, Xi’an 710064, China

**Keywords:** inverted asphalt pavement, rut prediction, model calibration, shift function, nonlinear incremental recursive principle, accelerated pavement testing

## Abstract

This study investigates the establishment and calibration method of the rut depth (RD) prediction model of inverted asphalt pavements (IAPs), based on full-scale accelerated pavement testing (APT), which facilitates the accurate and reliable design or maintenance of IAPs. A power function is adopted for the prediction model construction of the rut progression before the failure stage, based on the typical permanent deformation progression curve of flexible pavements. The APT loading history is divided into units, according to the difference in physical conditions, providing the basis for a cumulative RD analysis and model calibration. The nonlinear incremental recursive (IR) principle is applied in the RD analysis to consider the influence of the nonlinear material property, performance deterioration, and loading history on the RD development. Further, the rut shift function relating prediction models obtained from laboratory tests and full-scale APT is established to introduce the APT data in the calibration process. Accordingly, the mechanistic-empirical RD prediction model calibration method, based on APT and the IR principle, is proposed and applied in a case study of a IAP RD prediction model calibration. Four 3.5 m × 4 m IAP test sections S1–S4 are constructed and instrumented and 700,000- and 900,000-wheel loads are applied on test sections S1–S2 and S3–S4, respectively, using the heavy vehicle simulator. The test data from the different APT load units are utilized for the model calibration, and the resultant prediction errors range from −2.16 mm to 1.18 mm. The calibrated model can also be used for the RD prediction of IAPs with other design schemes, by updating the corresponding material-related coefficients and the finite element model, which is essential for the design and maintenance of IAPs. The proposed calibration method could be a useful reference for the establishment of flexible pavement performance prediction models.

## 1. Introduction

Rutting is a common distress frequently leading to structural failure and traffic safety issues for flexible pavements, and the inverted asphalt pavement (IAP) is especially prone to experience rutting, owing to the relatively weak support of the unbound aggregate base [1,2]. Generally, rutting is defined as the longitudinal surface depression along the wheel path and is characterized by the parameter of the rut depth (RD) [3,4]. Surface water, such as rainwater, tends to accumulate in the rutted portion of the pavement, which makes steering difficult and results in the hydroplaning of vehicles. Additionally, a reduced asphalt concrete (AC) layer thickness in the rutted portion may accelerate the progression of fatigue cracking and other pavement failures. To help highway agencies with proper work planning and budget allocation, accurate and reliable RD prediction models are necessary for the determination of desirable pavement design schemes and maintenance times or measures.

Many types of rut prediction models have been developed since the AASHO Road Test, in the late 1950s, during which large numbers of test data were available for model establishment [5]. At the early stage, laboratory specimen test results are normally used to build the empirical rut prediction models [6,7]. However, lab tests illustrate obvious differences, compared with field tests, regarding the size effect, boundary condition, and stress state, making the empirical models difficult to meet the accuracy requirement of engineering practice. Therefore, field projects, such as West Track, Mn/ROAD, and in-service pavements, were gradually developed and utilized to build the rut prediction model under real engineering application conditions [8,9,10,11]. Mechanistic-empirically (M-E) based accumulative permanent strain or deformation prediction models were established with variables, such as the number of load repetitions, temperature, layer stiffness, layer thickness, vertical strains, and shear strains [12,13]. The models are usually constructed and regressed, based on laboratory test results, then verified or calibrated with field test results [8,9,14]. However, complicated traffic load distribution and environment condition variations of the filed pavements require simplified assumptions for the verification or calibration phases, which affects the reliability of the acquired models.

The accelerated pavement testing (APT) technique emerges as the research requires, which aims to apply continuous wheel loads on full-scale test sections under controlled load and environmental conditions, until the structure fails within a short period of time [15,16]. Researchers from Australia [17], South Africa [18], the United States [19], France [20], Brazil [21], Costa Rica [22], and so on, have contributed a lot to the field over the last decades. Research results can be found in the long-term performance evaluations [23,24,25,26] and distress mechanism investigations [27,28] of pavement materials and structures, based on the APT technique, whereas there are lacking studies on the calibration methods of rut prediction models under complex test conditions, based on APT. Hence, calibrated M-E models, recommended in the design guide or provided by similar projects, are commonly used for IAPs in current research and engineering practice [29,30], which may not be applicable to the variety of pavement material types, structural combination, environment, and traffic characteristics of the local projects. Moreover, an accumulative RD analysis and prediction model construction generally utilizes Miner’s law [31], in which the linear superposition principle of this theory fails to include the impacts of the pavement material’s nonlinearity and loading history on the rut progression.

This study develops the calibration method of the RD prediction model for asphalt pavements, based on full-scale APT under complex loading conditions. The approach of introducing the APT, resulting in the calibration process and the rut analysis principle, and considering the effects of the material’s nonlinearity, test condition variations, and loading history on the rut accumulation, are investigated. Utilizing the test data obtained from APT of four IAP test sections using a heavy vehicle simulator (HVS), this study demonstrates and validates the proposed calibration method, and establishes the RD prediction model of IAPs under a full-scale APT condition.

## 2. Establishment of the Rut Prediction Model, Based on APT

### 2.1. Nonlinear Incremental Recursive Principle

The purpose of a highway pavement rutting analysis is to reveal the relation between the number of load repetitions and the accumulative RD, to establish proper RD prediction models. Miner’s law has been widely utilized for the calculation of accumulative pavement damage or distress, which linearly summates all of the structural damage generated in different load units under various physical conditions (such as stress level, temperature, water content, etc.…). The calculation method is shown in Equation (1).

(1) $$RD=\sum_{i=1}^{k}\sum_{j=1}^{m}PD_{ij}$$

where *RD* is the accumulative rut depth; *k* is the number of load units in the analysis period; *m* is the number of physical conditions in the *i*th load unit; and *PD_ij_* is the permanent deformation generated under the *j*th physical condition in the *i*th unit.

**Figure 1:**
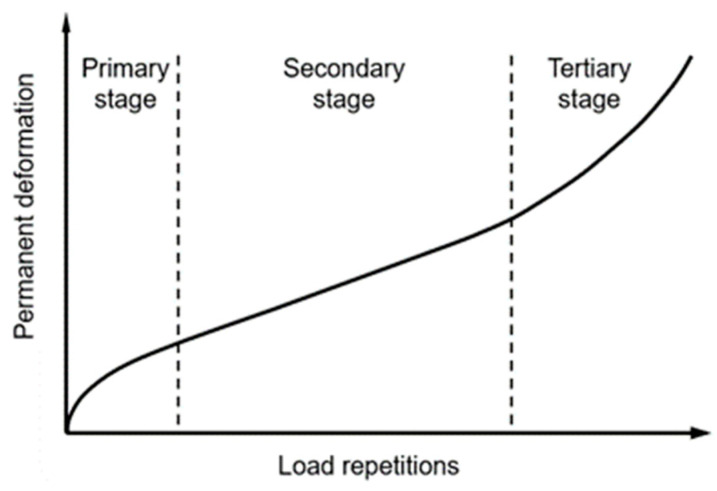
Typical rut progression curve of the asphalt concrete course.

According to Equation (1), Miner’s law assumes that the pavement’s permanent deformation (PD) increases linearly as the load repetitions increase under specific physical conditions. Accordingly, the PD formed in the different load units can be linearly added up to calculate the accumulative RD without considering the order of the units. However, as shown in Figure 1, the asphalt concrete course rut progression typically comprises three stages and demonstrates a nonlinear relation with the load repetitions. In the primary stage, the local plastic deformation is prone to be generated due to the stress concentration caused by the traffic loads around the internal voids and the micro damages after construction, resulting in the rapid increase of the permanent deformation. The high-density energy stored near the internal defects of pavement materials is released through the plastic deformation and defect expansion, resulting in the energy balance and reaching a relatively stable deformation state, in the secondary stage. Finally, at the tertiary stage, the structural resistance will drop close to the external load, owing to the development of micro damages and accumulated shear distortion. The permanent deformation will increase dramatically afterwards, causing the failure of the pavement.

**Figure 2:**
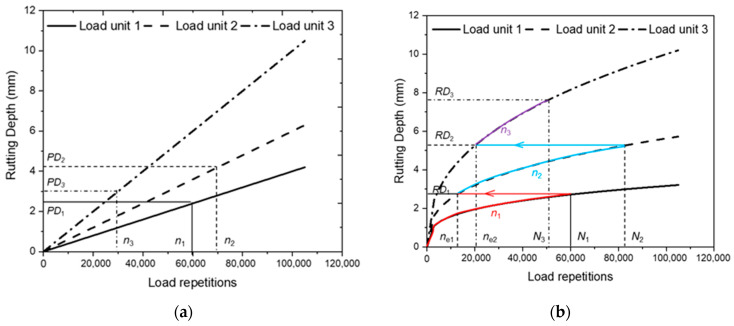
Accumulative rut depth calculation, based on the different principles: (**a**) Miner’s law; (**b**) incremental recursive method. Notes: *N*_1_ = *n*_1_, *N*_2_ = *n*_e1_ + *n*_2_, *N*_3_ = *n*_e2_ + *n*_3_.

According to the rut progression characteristics shown in Figure 1, the rut increasing rate varies continuously as the load repetition increases. Therefore, the order of load units will significantly affect the calculation result of the cumulative RD. Figure 2 illustrates the accumulative RD calculation of three load units in the order of 1, 2, and 3, using Miner’s law and incremental recursive (IR) principle. Compared with Miner’s law, the IR method is featured by calculating the rutting equivalent load repetitions (RELR) *n*_e_ to recurse the accumulation process to the next load unit, making it possible to consider the rut development laws in the different stages. Parameters, such as the layer stiffness, temperature, and pavement responses of the current unit will be updated during the recursive process. Accordingly, the calculation method could better replicate the rutting progression process of the pavement. In contrast, Miner’s law method directly sums up the predicted PD of all load units, which may well overestimate the cumulative RD, according to Figure 2a. Therefore, the IR method is used for the RD model calibration in the study.

Figure 2b illustrates the use of the nonlinear IR method in calculating the cumulative RD. Firstly, the RD at the end of load unit 1 (*RD*_1_) can be determined, according to the nonlinear RD prediction model with the load repetition *n*_1_. Then, the RELR *n*_e1_ producing *RD*_1_ under the rut development law of load unit 2, will be determined to recurse the calculation process to unit 2. Thereafter, the accumulative load repetition *N*_2_ (i.e., *n*_e1_ + *n*_2_) at the end of unit 2 and the corresponding RD (*RD*_2_), can be determined with the nonlinear prediction model. A similar process of the recursing RD analysis to the next load unit with RELR and calculating the next incremental PD, will be repeated until the last unit of the analysis period, in order to obtain the final cumulative RD.

### 2.2. Establishment of the Rut Prediction Model, Based on the Lab Test

A comprehensive rut prediction model should be able to predict all three stages of the rut progression of typical flexible pavements. However, fatigue cracking can sometimes precede rutting and maintenance measures will usually be taken to restore the pavement’s serviceability before the tertiary stage, which makes the modeling of tertiary stage rutting not necessary [6]. Therefore, the calibration of the RD prediction model is mainly focused on the primary and secondary stages in the study, and the power function shown in Equation (2) is adopted for the model establishment. It can be inferred from the power function curve features, that power index *b* mainly determines the shape of the curve, while coefficient *a_i_* indicates the height of the curve. Therefore, coefficients *a_i_* and *b* are supposed to be related to the loading conditions (such as the load level, loading rate, temperature, etc.…) and the inherited material property, respectively.

(2) $$RD_{i}=a_{i}\times N_{i}^{b}=a_{i}\times {\left(n_{e,i-1}+n_{i}\right)}^{b}$$

where *RD_i_* and *N_i_* are the accumulative rut depth in mm and the accumulative axle loads at the end of the *i*th load unit; *n_i_* is the axle loads in the *i*th load unit; *n*_e,*i*−1_ is the rutting equivalent axle loads producing *RD_i_*_−1_ under the loading and physical conditions of the *i*th load unit, which can be calculated according to Equation (3), as follows:

(3) $$n_{e,i-1}={\left(RD_{i-1}/a_{i}\right)}^{1/b}$$

where *a_i_* and *b* are the loading condition related and material property related model regression coefficients, respectively.

The M-E design approach generally determines the initial form of the asphalt pavement performance prediction models through lab specimen testing, and verifies or calibrates the model, based on the field test or APT results afterward. The initial form of the prediction model, shown in Equation (2), is determined through the asphalt mixture SPT dynamic creep test (i.e., the flow number test) in the study. The material-related coefficient *b* is stable or constant for a certain type of AC mixture, and can be determined in the lab test. Coefficient *a* representing the increasing speed of the permanent deformation is significantly affected by the test temperature and loading conditions in the creep test. Therefore, the variable temperature *T* is used to characterize *a* with the nonlinear model presented in Equation (4). However, considering the stress state difference between the lab and field tests, the effects of the loading conditions are better investigated in the following calibration with full-scale APT results. Consequently, variables relating to the loading conditions are not included in Equation (4).

(4) $$a=k\times {\left(\frac{T}{T_{ref}}\right)}^{c}$$

where *T* is the lab test temperature; *T*_ref_ is the reference temperature to normalize the model dimension, whose value is usually adopted as 20 °C; *k* and *c* are undetermined model coefficients.

### 2.3. Establishment of the Shift Function and Prediction Model, Based on the Full-Scale APT

It is noteworthy that the size effect, stress state, and boundary conditions of small-scale specimen tests are significantly different from those of the pavement structures in the field. To obtain the RD prediction models with a higher accuracy and reliability for actual projects, it is necessary to establish the shift function converting the rut prediction model from the lab test to the full-scale test condition using full-scale APT. The basic shift relation between the predicted RD from lab test and full-scale APT is illustrated in Equation (5).

(5) $$RD_{\mathrm{APT}}=RSF\times RD_{\mathrm{lab}}=RSF\times aN^{b}$$

where *RD*_APT_ and *RD*_lab_ are the predicted rut depth, based APT and lab test in mm, respectively; *RSF* is the rut shift factor; *N* is the accumulative axle loads.

The more specific composition of RSF can be determined, based on the primary differences between the lab test and full-scale APT related to the rutting development. The AC dynamic elastic modulus is able to represent the size effect and stress state of the layer, and the maximum vertical compressive strain on top of the subgrade could reflect the lateral restraints and overall stiffness of the whole pavement structure. Accordingly, these two parameters that are closely related to the pavement rutting are utilized to establish the shift function shown in Equation (6).

(6) $$RSF=d\times {\left(\frac{E_{a}}{E_{a,\mathrm{ref}}}\right)}^{e}{\left(\frac{\varepsilon_{v}}{\varepsilon_{v,\mathrm{ref}}}\right)}^{f}$$

where *E_a_* is the dynamic elastic modulus of the AC layer in MPa; ε*_v_* is the maximum vertical compressive strain on top of the subgrade in 10^−6^; *E_a_*_,ref_ and *ε_v_*_,ref_ are the reference modulus and vertical compressive strain to normalize the model dimension; *d*, *e,* and *f* are undetermined model coefficients.

Finally, the RD prediction model of the AC layer established, based on full-scale APT can be obtained by combining Equations (4)–(6) as Equation (7). To finalize the undetermined model coefficients, APT of the full-scale asphalt pavement test sections under complex loading conditions are required for the calibration of the shift function. Methods of APT setup, model parameter determination, and model calibration, based on full-scale APT, are demonstrated as follows.

(7) $$RD_{\mathrm{APT}}=k\times d\times {\left(\frac{T}{20}\right)}^{c}{\left(\frac{E_{a}}{E_{a,\mathrm{ref}}}\right)}^{e}{\left(\frac{\varepsilon_{v}}{\varepsilon_{v,\mathrm{ref}}}\right)}^{f}N^{b}$$

## 3. Full-Scale APT Setup and Model Parameters Determination

### 3.1. Full-Scale APT Setup and Loading History Division

#### 3.1.1. Full-Scale APT of Inverted Asphalt Pavement Test Sections

**Figure 3:**
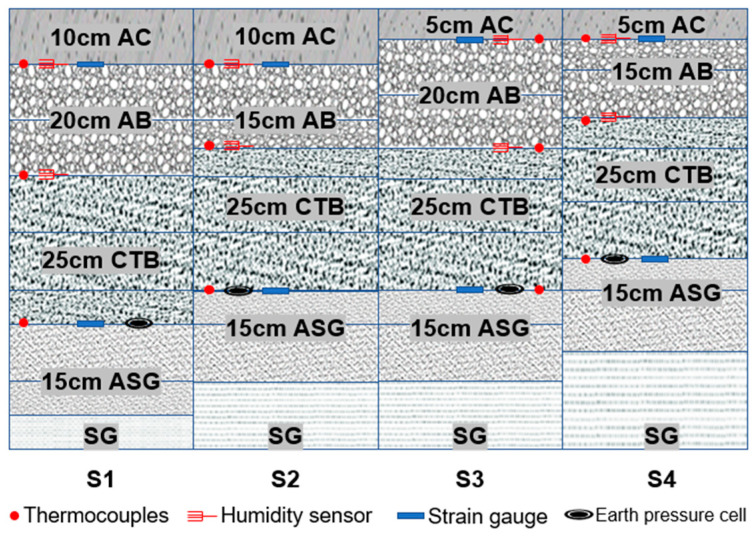
Structure combination and instrumentation design scheme of APT sections. Note: AC, AB, CTB, ASG, and SG denote the asphalt concrete, aggregate base, cement treated base, aggregate subgrade, and subgrade, respectively.

Four full-scale IAP test sections S1-S4 are constructed to facilitate the RD prediction model calibration. The structure combination scheme is determined by referring to the typical high-class highway pavement structure combinations in the South African flexible pavement design guide, to reduce the AC layer thickness and prevent the reflection cracks of semi-rigid base asphalt pavements. The test sections are instrumented with thermocouples, water content sensors, strain gauges, and an earth pressure cell to obtain the environmental condition and dynamic response data. The structure combination and instrumentation design scheme of test sections S1–S4 are shown in Figure 3. The data acquisition system DH3820 [32,33] is used for the data collection. The dynamic response data is sampled and stored at the frequency of 100 Hz, while the temperature and water content data are monitored on an hourly basis during APT.

**Figure 4:**
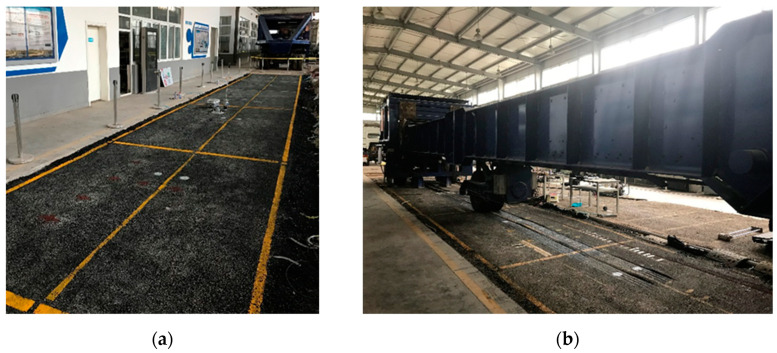
Test sections and accelerated pavement test loading facility: (**a**) test sections; (**b**) APT loading facility.

APT is conducted in an indoor laboratory to characterize the life cycle rutting performance of the test sections. Each test section is 3.5 m wide and 4 m long, and HVS (version VI-A) is utilized to apply 40–70 kN and 9 km/h moving loads on the test sections, using the dual-wheel carriage. The HVS trafficking is in a bi-directional mode without lateral wanders, and 700,000 and 900,000 load repetitions are applied on test sections S1–S2 and S3–S4, respectively. The test sections and APT loading facility are shown in Figure 4.

Falling weight deflectometer (FWD) testing is conducted on the test sections after every 20,000 HVS load repetition at the prespecified positions. In the FWD test, 50 kN (707 kPa) load is applied on the pavement by dropping the standard weight on a 30 cm diameter rigid bearing plate. The FWD test data is used for the moduli back calculation of the pavement layers during the APT process.

#### 3.1.2. APT Loading History Division

The prediction model shown in Equation (7) indicates that the cumulative RD is influenced by the parameters of the test temperature, AC layer dynamic modulus, and compressive strain on top of the subgrade. To facilitate the determination of the related model coefficients in the calibration, different load levels and test temperatures are designed for different stages of APT. The rutting analysis utilizing the nonlinear prediction model should be conducted within the individual load unit with relatively stable conditions of the axle load level and test temperature. Consequently, the HVS APT loading history is divided into different load units; 50 kN half axle duel-wheel load is set for 0–700,000 load repetitions of test sections S1 and S2. However, due to the significant temperature change from 0–29,000 to 30,000–700,000 load repetitions, the loading history of S1/S2 is divided into two load units, as S1-1/S2-1 and S1-2/S2-2. The half axle duel-wheel loads of 40 kN, 50 kN, 55 kN, 60 kN, and 70 kN are adopted for the HVS loading of test sections S3 and S4. Accordingly, the loading history of S3 and S4 is divided into seven load units as S3-1–S3-7 and S4-1–S4-7, respectively. The APT loading history division results of the four test sections for the rutting analysis are presented in Table 1.

### 3.2. Model Parameters and Coefficients Determination

#### 3.2.1. Representative Temperature of the AC Layer for the Rutting Analysis

The temperature in the AC layers significantly affects the rutting development, owing to the temperature susceptibility property of the asphalt mixtures. However, different from the controlled temperature condition in the lab tests, the temperature in the AC layer varies with time following the ambient temperature variation. To calibrate the proposed RD prediction model for the different load units, the representative AC temperature during one load unit needs to be properly adopted. Since the APT in this study is conducted indoors, the temperature at the middle depth of the AC layer is relatively stable and is able to represent the average level of the temperature distribution along the whole depth of the layer. Therefore, the average value of the temperature at the middle depth of the AC layer during one load unit is adopted as the representative temperature for the rutting analysis. The middle depth temperature is calculated approximately as the average of the temperatures at the bottom and on the surface of the AC layer. The AC layer’s representative temperature can be calculated, based on the hourly monitored data from the instrumented thermocouples in the test sections.

#### 3.2.2. Representative Moduli of the Pavement Courses and the Maximum Compressive Strain on Top of the Subgrade

The moduli of different pavement courses are determined by the back calculation of the FWD test results when calibrating the model with APT data. The average of the back-calculated moduli of two adjacent FWD tests is adopted as the representative moduli for the different courses. Unlike the courses insusceptible to temperature, the AC layer moduli back calculated under different temperatures should be converted into the corresponding 20 °C equivalent dynamic modulus, according to Equation (8). The conversion function is obtained by the regression of dynamic modulus test results of the asphalt mixture used in the test sections at temperatures of 5 °C, 20 °C, 35 °C, 45 °C, and 54 °C. Accordingly, the average 20 °C equivalent dynamic modulus of the back-calculated moduli from two adjacent FWD tests is adopted as the representative AC layer modulus.

(8) $$E_{a}=E_{T}\times e^{0.0775\left(T-20\right)}$$

where *E_a_* is the 20 °C equivalent dynamic modulus of the AC layer in MPa; *E_T_* is the AC layer dynamic modulus derived from the FWD back calculation at the temperature of *T* °C in MPa.

**Figure 5:**
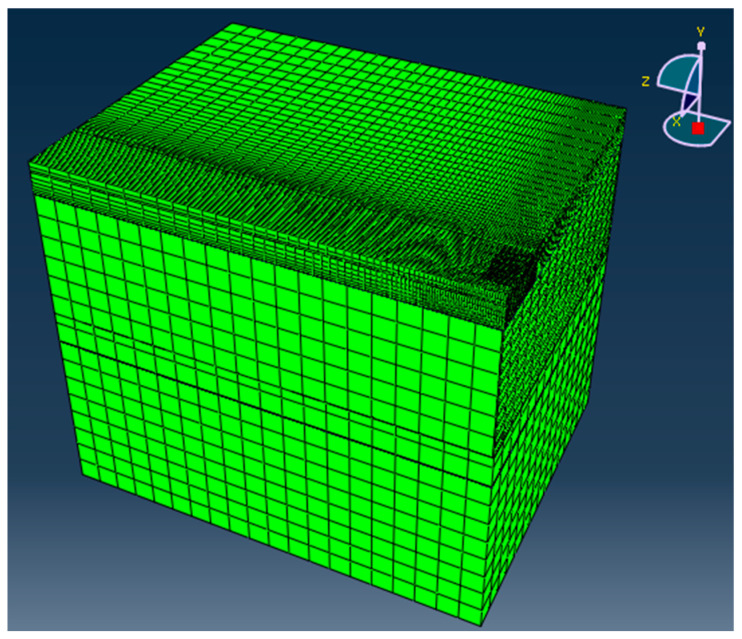
The geometry and meshing result of the finite element model utilized in the study.

The 3D finite element (FE) model considering cracks in the cement stabilized subbase and the plastic deformation property of the unbound aggregate base, was established by the authors [34]. The geometry and meshing result of the FE model are illustrated in Figure 5. The FE model is used to calculate the maximum compressive strain on top of the subgrade, according to the structure combination and representative layer modulus of the test sections in the different load units. The thickness reduction of the AC layer caused by the rut depression is considered in the modulus back calculation and FE modeling of the test sections.

#### 3.2.3. Material Property and Temperature Related Coefficient Calibration

The flow number test is utilized to calibrate the material and temperature related coefficients in Equation (2). One Hz repeated half vector wave axial load is applied on the specimen at the specified temperature, by loading for 0.1 s and unloading for 0.9 s afterward. The axial permanent strain and its variation rate are measured during the loading process. The loading times corresponding to the point with the smallest strain variation rate is determined as the flow number. In this study, core specimens are drilled from test sections, and the flow number tests are conducted at temperatures of 20 °C, 35 °C, and 45 °C. Three parallel specimens are set for each temperature condition, which are conditioned at the target temperature for 8 h and loaded under the deviator stress of 600 kPa afterward.

According to the test results, the prediction model shown in Equation (2) is fitted using the axial permanent deformation and loading times of the specimens tested at different temperatures. The average model regression results are shown in Table 2, indicating that the material related coefficient *b* can be determined as 0.4945 and coefficient *a* increases as the test temperature increases. Therefore, *a* can be further determined, based on Equation (4) utilizing the regression results at different temperatures. The fitting results of *a* are presented in Table 2, and Equation (4) can be determined as Equation (9), accordingly.

(9) $$a=0.2592\times {\left(\frac{T}{20}\right)}^{0.9915}$$

### 3.3. Rut Progression History of the APT Sections

**Figure 6:**
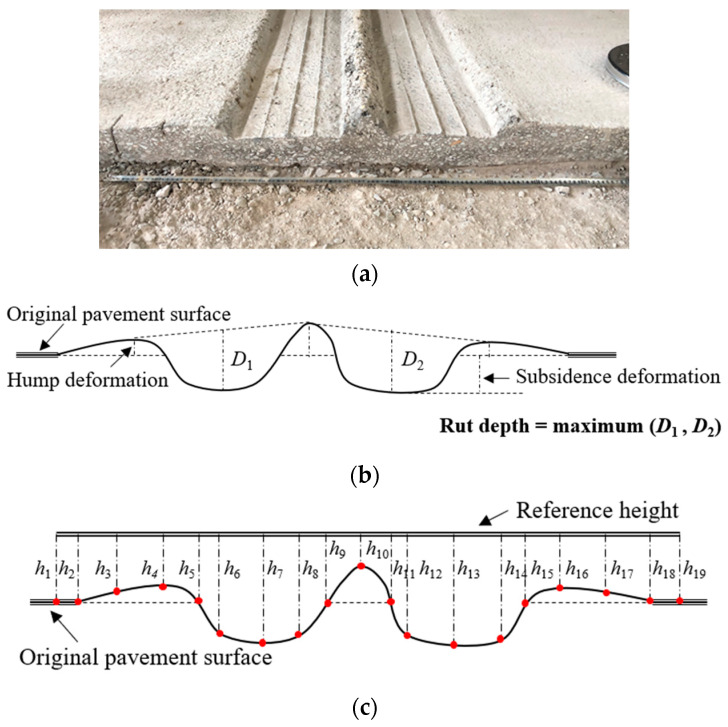
The APT rut deformation result, rut depth definition, and rutting profile measurement method: (**a**) AC layer trench excavation result after APT; (**b**) Rut depth definition; (**c**) Rutting profile measurement method.

Asphalt pavement rutting can be categorized into three types, depending on the root cause, i.e., the mix rutting, subgrade rutting, and densification. According to the APT results, rutting of the four test sections primarily occurred in the AC layer, as shown in Figure 6a. The typical rutting profile is shown in Figure 6b which is mix rutting caused by the AC mixture shear distortion under repeated loads. The RD measurements are conducted on the test sections after every 20,000 HVS load repetition, at the prespecified positions. The RD is defined as the surface permanent deformation, illustrated in Figure 6b, and distances of 19 points along the rutting profile to the reference height, shown in Figure 6c, are measured to characterize the rutted cross-section profile and calculate the RD. Three cross sections, i.e., the cross sections at the beginning, the middle, and the end of the longitudinal loading wheel track of each test section, are selected for the RD measurement of the corresponding test section. The average of the RD measurements of the three cross sections is adopted as the representative RD value for the corresponding test section after every 20,000 load repetitions.

**Figure 7:**
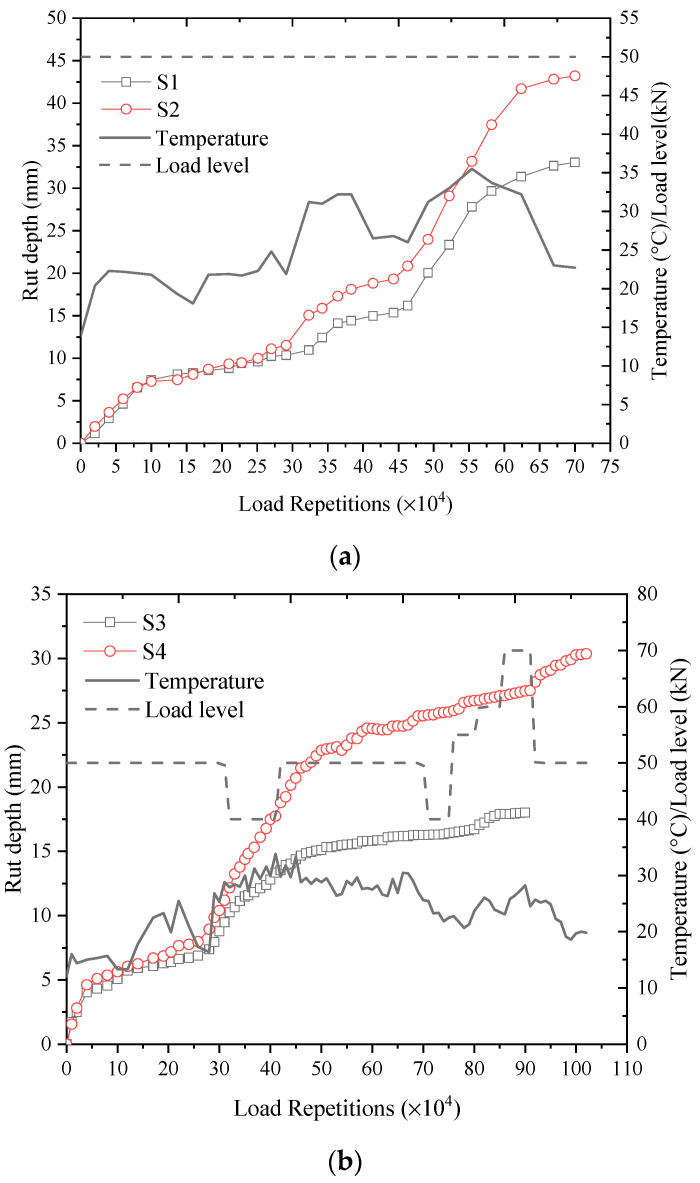
The rut progression history and corresponding test conditions of the four test sections: (**a**) rut progression history of test sections S1 and S2; (**b**) rut progression history of test sections S3 and S4.

Based on the measurement results during APT, the RD progression history, along with the corresponding AC layer, representative temperatures, and load levels of test sections S1–S4 are obtained and presented in Figure 7. It should be noted that the rut progression of test sections S1 and S2 entered the tertiary stage from the load repetition of 492,000, according to the typical flexible pavement rut progression curve. To ensure the validity of the model calibration results, load units S1-2 and S2-2 are divided into two subunits from the load repetition of 492,000, as S1-2-1, S1-2-2, and S2-2-1, S2-2-2, respectively. The APT data of units S1-2-2 and S2-2-2 will not be used for the model calibration in the study.

## 4. Calibration of the IAP Rut Prediction Model, Based on Full-Scale APT

To demonstrate the application method and feasibility of the proposed model calibration method for engineering practices, the RD prediction model of the IAP is calibrated using the full-scale APT results of the four test sections. The primary task of model calibration is to determine the prediction model shift function (Equation (6)) once the material and temperature related model coefficients were determined. The measured RD progression history, back-calculated layer moduli, representative layer temperature, and FE model calculated pavement responses of test sections S1-S4 during the APT, are used to calibrate and determine the model coefficients.

### 4.1. Calibration in the Initial Load Units

As the model calibration is based on a nonlinear incremental recursive method, the rutting equivalent load repetitions *n*_e,p_ need to be solved to recurse the calibration process from the current load unit to the next. However, the initial unit is calibrated without computing *n*_e,p,_ as no damage or deformation are accumulated before this. Therefore, the RD test results after the different load repetitions in the initial load units of S1, S2, S3, S4 are used to preliminarily calibrate the model shown in Equation (5). Coefficients *a* and *b* were determined according to the flow number tests, and *RSF* could be further calculated, based on the regression results, which is presented in Table 3. Thereafter, the coefficients in Equation (6) can be regressed using the back-calculated layer moduli and computed maximum compressive strains in the initial load units, illustrated in Table 4. Accordingly, the RSF function and RD prediction model can be initially determined as Equations (10) and (11), respectively. 

However, the RSF is significantly affected by the pavement structure dimensional effect, boundary condition, stress state, axle load level, and ambient temperature compared to the lab tests. The APT data of the full-scale test sections under a more diversified load and temperature conditions is necessary for more precise and reliable model calibration results. Therefore, RSF will be further calibrated utilizing the test data from the consecutive load units of S1–S4.

(10) $$RSF=0.1023\times {\left(\frac{E_{a}}{700}\right)}^{-0.0848}{\left(\frac{\varepsilon_{v}}{12}\right)}^{0.2599}$$

(11) $$RD=RSF\times a{\left(n_{e,p}+n\right)}^{b}=0.0265\times {\left(\frac{E_{a}}{700}\right)}^{-0.0848}{\left(\frac{T}{20}\right)}^{0.9915}{\left(\frac{\varepsilon_{v}}{12}\right)}^{0.2599}\;N^{0.4945}$$

### 4.2. Calibration in the Consecutive Load Units

Various ambient temperature conditions and 40–70 kN half axle dual-wheel loads are set in the consecutive HVS APT load units of test sections S1–S4. The test data of those load units can be used to further calibrate the prediction model. Taking test section S3 as an example, the model calibration method and process, based on the APT results and the nonlinear IR principle are described, as follows. The calibration of the consecutive load units of other test sections is similar to that of S3.

#### 4.2.1. Model Calibration in the Second Load Unit

Half axle load of 40 kN and the representative temperature of 30.2 °C at the middle depth of the AC layer are adopted in the load unit S3-2. The rutting growth law will change, owing to the change of the load level, temperature, and pavement responses, compared to the previous load unit. Hence, to recurse the calibration process from S3-1 to S3-2, the RELR *n*_e1_ completing the RD of S3-1 with the rutting growth law in unit S3-2 needs to be solved, firstly. *n*_e1_ can be calculated, according to Equation (12) using the representative layer moduli, representative AC temperature, and maximum compressive strain in unit S3-2, as well as the RD at the end of unit S3-1. The average of all measured results in unit S3-2 are adopted as the representative layer moduli and temperature. Then, the maximum compressive strain on top of the subgrade can be computed, based on the representative layer moduli, layer thicknesses, and axle load level using the formerly developed FE model [34]. The parameters determination method is the same for other load units and the parameter calculation results of the consecutive load units are presented in Table 5.

(12) $$n_{e1}={\left(\frac{RD_{1}}{RSF\times a_{2}}\right)}^{1/b}={\left(\frac{RD_{1}}{0.0265\times {\left(\frac{E_{a2}}{700}\right)}^{-0.0848}{\left(\frac{T_{2}}{20}\right)}^{0.9915}{\left(\frac{\varepsilon_{v2}}{12}\right)}^{0.2599}}\right)}^{1/0.4945}$$

where *RD*_1_ is the accumulative rut depth at the end of load unit S3-1; *E_a_*_2_, *T*_2_, and *ε_v_*_2_ are the representative modulus and temperature of the AC layer and the representative maximum compressive strain on top of the subgrade in load unit S3-2, respectively.

The equivalent cumulative load repetition (*n*_e1_ + *n*) in load unit S3-2 can be obtained after *n*_e1_ is determined as 135,869. Subsequently, the prediction model regression analysis can be conducted, according to Equation (13) utilizing the measured RD data and representative AC layer temperature after the different load repetitions in load unit S3-2. The regressed *RSF* and the corresponding regression determination coefficient are 0.0694 and 0.9291, respectively, which are documented in Table 5.

(13) $$RD=RSF\times a_{2}{\left(n_{e1}+n\right)}^{0.4945}=RSF\times 0.2592\times {\left(\frac{T_{2}}{20}\right)}^{0.9915}{\left(n_{e1}+n\right)}^{0.4945}$$

#### 4.2.2. Model Calibration in the Third Load Unit

The third load unit S3-3 adopts the 50 kN dual-wheel load and 28.0 °C representative AC layer temperature. Firstly, the RELR *n*_e2_ corresponding to the cumulative RD of load unit S3-2 is calculated using Equation (14). The parameters used in the calculation can be found in Table 5.

(14) $$n_{e2}={\left(\frac{RD_{2}}{0.0265\times {\left(\frac{E_{a3}}{700}\right)}^{-0.0848}{\left(\frac{T_{3}}{20}\right)}^{0.9915}{\left(\frac{\varepsilon_{v3}}{12}\right)}^{0.2599}}\right)}^{1/0.4945}$$

where *RD*_2_ is the accumulative rut depth at the end of load unit S3-2; *E_a_*_3_, *T*_3_, and *ε_v_*_3_ are the representative modulus and temperature of the AC layer and the representative maximum compressive strain on top of the subgrade in load unit S3-3, respectively.

The calculation result of *n*_e2_ is 351,161, and the equivalent cumulative load repetition in load unit S3-3 can be updated as (*n*_e2_ + *n*) afterward. Thereafter, the regression analysis of the RD prediction model is carried out according to Equation (15) using the measured RD and representative AC layer temperature after different load repetitions in unit S3-3. The calculated *RSF* and the corresponding regression determination coefficient are 0.0663 and 0.9502, respectively.

(15) $$RD=RSF\times a_{3}{\left(n_{e2}+n\right)}^{0.4945}=RSF\times 0.2592\times {\left(\frac{T_{3}}{20}\right)}^{0.9915}{\left(n_{e2}+n\right)}^{0.4945}$$

#### 4.2.3. Model Calibration in the *i*th Load Unit

Firstly, the rutting equivalent load repetition *n*_e,*i*−1_ corresponding to the cumulative *RD* of load unit S3-(*i*−1), should be calculated according to Equation (16). The parameters involved in the calculation can be found in Table 5.

(16) $$n_{e,i-1}={\left(\frac{RD_{i-1}}{0.0265\times {\left(\frac{E_{ai}}{700}\right)}^{-0.0848}{\left(\frac{T_{i}}{20}\right)}^{0.9915}{\left(\frac{\varepsilon_{vi}}{12}\right)}^{0.2599}}\right)}^{1/0.4945}$$

where *RD_i_*_−1_ is the accumulative rut depth at the end of load unit S3-(*i*−1); *E_ai_*, *T_i_*, and *ε_vi_* are the representative modulus and temperature of the AC layer and the representative maximum compressive strain on top of the subgrade in load unit S3-*i*, respectively.

The rutting equivalent cumulative load repetitions in load unit S3-*i* could be updated as (*n*_e,*i*−1_ + *n*) once *n*_e,*i*−1_ is determined. Subsequently, the RD prediction model could be regressed, according to Equation (17) using the measured RD and representative AC layer temperature after different load repetitions in load unit S3-*i* to determine the *RSF*.

(17) $$RD_{i}=RSF\times 0.2592\times {\left(\frac{T_{i}}{20}\right)}^{0.9915}{\left(n_{e,i-1}+n\right)}^{0.4945}$$

The total number of load units can be determined, based on the loading history division results of the different test sections. According to the above calibration method, the test data from other consecutive load units of S1–S4 can be utilized to further calibrate the *RSF*, and the corresponding calibration results are shown in Table 6. Then, the shift function can be calibrated, according to Equation (6). However, the AC layer thickness is found to have a significant influence on the rutting development [35]. Therefore, the item of the AC layer thickness ha is added in the shift function, and the measured AC layer thicknesses of test sections S1–S4 during the APT are used for calibration. The parameter calculation results and *RSF* calibration results using test data from the consecutive load units of S1–S4 are presented in Table 6.

**Figure 8:**
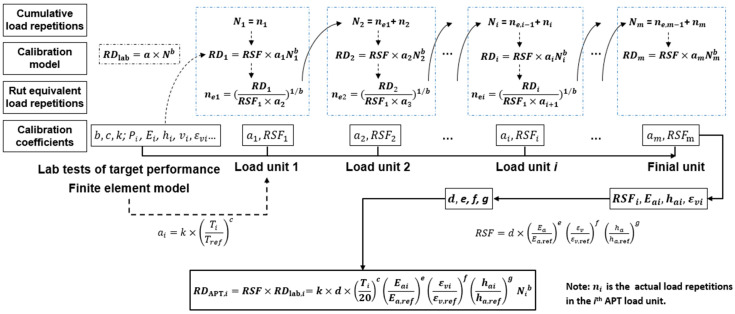
Calibration method and process of the rut depth prediction model, based on the full-scale APT.

Consequently, the shift function shown in Equation (18) can be obtained after the model calibration using the APT data of the four full-scale IAP test sections, and the prediction model of the RD at the end of the *i*th APT load unit can be determined as Equation (19). The calibration method and process can be illustrated as the diagram shown in Figure 8. The calibration method is developed, based on the results of the lab test and multi-units full-scale APT utilizing the nonlinear IR principle. By altering the lab test and APT setup, the method and process illustrated in Figure 8 could be used for the establishment and calibration of other performance prediction models of asphalt pavements.

(18) $$RSF=0.1491\times {\left(\frac{E_{a}}{700}\right)}^{-0.1738}{\left(\frac{\varepsilon_{v}}{12}\right)}^{0.2018}{\left(\frac{h_{a}}{50}\right)}^{0.1269}$$

(19) $$RD_{APT,i}=0.0386\times {\left(\frac{Ti}{20}\right)}^{0.9915}{\left(\frac{E_{ai}}{700}\right)}^{-0.1738}{\left(\frac{\varepsilon_{vi}}{12}\right)}^{0.2018}{\left(\frac{h_{ai}}{50}\right)}^{0.1269}\;{\left(n_{e,i-1}+n_{i}\right)}^{0.4945}$$

where *RD*_APT,*i*_ is the predicted accumulative rut depth at the end of the *i*th load unit; *T_i_*, *E_ai_*, *h_ai_*, and *ε_vi_* are the representative temperature, modulus, and thickness of the AC layer, and the representative maximum compressive strain on top of the subgrade in the *i*th load unit, respectively; *n*_e,*i*−1_ is the rutting equivalent load repetition corresponding to *RD*_APT,*i*−1_; *n_i_* is the load repetition in the *i*th load unit.

### 4.3. Evaluation of the Calibrated RD Prediction Model

**Figure 9:**
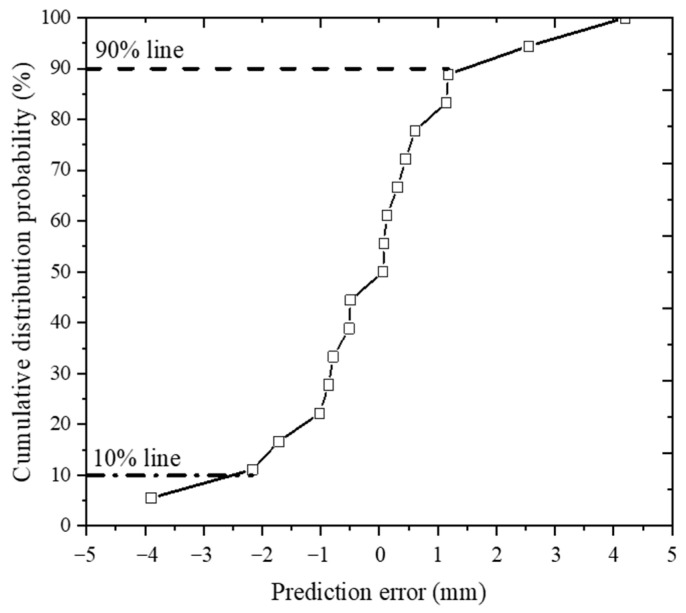
Cumulative probability distribution curve of the rut depth prediction error of all load units.

To evaluate the validity and reliability of the calibrated RD prediction model shown in Equation (19), the cumulative RD at the end of the different load units of test sections S1–S4 are predicted, based on the representative temperature, representative layer moduli, AC layer thickness at the beginning of the unit, and the representative FEM computed maximum compressive strain on top of the subgrade of the different load units. The parameters utilized in the calculation of the predicted cumulative RD can be found in Table 3, Table 4, Table 5 and Table 6. The predicted cumulative RD results are compared with the corresponding measured data, and the prediction errors are determined and illustrated in Table 7, which ranges from −3.9 to 4.2 mm. Moreover, the cumulative probability distribution curve of the prediction errors presented in Figure 9 shows that 80% of the errors are in the range of −2.16–1.18 mm, indicating relatively stable and reliable prediction results of the calibrated model. Therefore, the calibrated RD prediction model could be utilized for the design and maintenance of IAPs. The proposed calibration method of the RD prediction model could be a useful reference for the determination of the asphalt pavement performance prediction models.

## 5. Conclusions

The study aims to develop the calibration method for the rut prediction model of IAPs, based on full-scale APT. The nonlinear IR principle was used for the accumulative RD analysis, and a power type RD prediction model was established, according to the typical rut progression law of flexible pavements. The shift function of the RD prediction model from the lab specimen tests to full-scale APT conditions was developed to introduce the full-scale pavement performance test data to the calibration process. Four full-scale IAP test sections were constructed and instrumented to facilitate the model calibration. The APT with 700,000 and 900,000 repetitions of HVS loads were applied on test sections S1–S2 and S3–S4, respectively. The RD measurements and FWD tests were carried out on the test sections after every 20,000 load repetitions. The RD prediction model of IAPs was calibrated using the measured APT data, illustrating the prediction error mainly ranging from −2.16 mm to 1.18 mm. The conclusions obtained from the model calibration process are listed as follows:

(1) Compared with the traditional linear permanent deformation analysis method based on Miner’s law, the IR principle could better consider the impact of the pavement material’s nonlinear property, complex loading history, and environment conditions on the RD accumulation. The IR based analysis method is conducive to improving the reliability and accuracy of the RD prediction by better replicating the rutting development process of the actual flexible pavements.

(2) The shift function linking the RD prediction models derived from the lab tests and field tests is essential for the model calibration based on APT, which can be constructed with parameters reflecting the primary difference of the different test conditions. The loading history division and model parameter determination methods provide the basis for the model calibration method, based on full-scale APT and the nonlinear IR principle. The proposed calibration method could be used as a reference for the establishment of the RD prediction model of flexible pavements.

(3) The RD prediction model of IAPs is calibrated using small-scale specimen tests and full-scale APT. The calibrated model can be used for the RD prediction of IAPs with other design schemes, by updating the material-related coefficients and the corresponding FE models, which is convenient for engineers to apply in various local projects.

(4) More IAP test sections with different design schemes will be constructed for HVS APT to further calibrate the RD prediction model obtained in the study, and the long-term performance data of the filed IAP will be collected to develop the shift function from full-scale APT to the filed in-service pavement condition.

## Figures and Tables

**Table 1:** APT loading history division results of the four test sections S1–S4 for the rutting analysis.

Test Section	APT Loading History	Load Repetition (×10^4^)	Axle Load (kN)	Load Unit ID
S1/S2	20 March 2018–7 May 2018	0–29	50	S1-1/S2-1
	22 June 2018–21 October 2018	30–70	50	S1-2/S2-2
S3/S4	26 February 2018–19 March 2018	0–29	50	S3-1/S4-1
	6 August 2019–18 August 2019	30–43	40	S3-2/S4-2
	18 August 2019–11 September 2019	44–70	50	S3-3/S4-3
	11 September 2019–16 September 2019	71–75	40	S3-4/S4-4
	17 September 2019–20 September 2019	76–80	55	S3-5/S4-5
	21 September 2019–25 September 2019	81–85	60	S3-6/S4-6
	26 September 2019–29 September 2019	86–90	70	S3-7/S4-7

**Table 2:** Fitting results of the rut depth prediction model coefficients, based on the asphalt mixture flow number test data.

*T* (°C)	*a*	*b*	*R* ^2^
20	0.26	0.4945	0.9999
35	0.45	0.4945	0.9033
45	0.58	0.4945	0.9761
Fitting results of *a*	*k* = 0.2592, *c* = 0.9915, *R*^2^ = 0.99		

**Table 3:** Initial calibration results of the rut depth prediction model coefficients, based on the test results in the initial load units.

Load Unit ID	*RD* (mm)	*RSF × a*	*b*	*R* ^2^	*T* (°C)	*a*	*RSF*
S1-1	10.35	0.0212	0.4945	0.9515	21.3	0.2759	0.0768
S2-1	11.52	0.0221	0.4945	0.9803	20.3	0.2631	0.0840
S3-1	8.77	0.0157	0.4945	0.9426	16.8	0.2181	0.0720
S4-1	10.4	0.0181	0.4945	0.9440	19.3	0.2502	0.0723

**Table 4:** Initial calibration data and results of the rut shift factor function, based on the test results in the initial load units.

Load Unit ID	*RSF*	*E_a_* (MPa)	*ε_v_* (10^−6^)
S1-1	0.0768	5989	8.7
S2-1	0.0840	6512	10.8
S3-1	0.0720	35,042	10.1
S4-1	0.0723	35,697	12.5
Fitting results of *RSF*	*d* = 0.1023; *e* = −0.0848; *f* = 0.2599; *R*^2^ = 0.8755		

**Table 5:** Calculation results of the parameters used for the model calibration and model regression results of the different load units.

Load Unit ID.	*RD* (mm)	*E_a_* (MPa)	*T* (°C)	*ε_v_* (10^−6^)	*a*	*RSF*	*R* ^2^
S1-2-1	15.4	13,919	29.1	8.6	0.3766	0.0836	0.9626
S2-2-1	19.3	10,987	28.2	9.5	0.3638	0.1008	0.9609
S3-2	13.9	22,962	30.2	6.6	0.3900	0.0694	0.9291
S3-3	16.3	21,711	28.0	8.4	0.36184	0.0663	0.9502
S3-4	16.4	22,604	24.0	6.9	0.31056	0.0660	0.9893
S3-5	16.7	18,846	22.3	9.4	0.2887	0.0716	0.9992
S3-6	17.9	20,243	23.7	10.3	0.3067	0.0751	0.996
S3-7	18.0	18,352	23.7	12	0.3067	0.0747	0.9822
S4-2	19.2	23,314	30.3	7.8	0.3913	0.0839	0.9621
S4-3	25.5	20,607	28.9	9.8	0.3734	0.0807	0.9492
S4-4	25.8	22,027	23.8	8.1	0.3080	0.0702	0.9816
S4-5	26.7	20,802	22.4	11.1	0.2900	0.0761	0.9915
S4-6	27.1	24,309	24.7	12	0.3195	0.0747	0.9927
S4-7	27.5	21,704	25.8	13.5	0.3336	0.0764	0.9732

**Table 6:** Parameter calculation results and *RSF* calibration results from the consecutive load units of S1, S2, S3 and S4.

Load Unit ID	*RSF*	*E_a_* (MPa)	*ε_v_* (10^−6^)	*h_a_* (mm)
S1-2-1	0.0836	13,919	8.6	89.7
S2-2-1	0.1008	10,987	9.5	88.5
S3-2	0.0694	22,962	6.6	41.2
S3-3	0.0663	21,711	8.4	36.1
S3-4	0.0660	22,604	6.9	33.7
S3-5	0.0716	18,846	9.4	33.6
S3-6	0.0751	20,243	10.3	33.3
S3-7	0.0747	18,352	12	32.1
S4-2	0.0839	23,314	7.8	39.6
S4-3	0.0807	20,607	9.8	30.8
S4-4	0.0702	22,027	8.1	24.5
S4-5	0.0761	20,802	11.1	24.2
S4-6	0.0747	24,309	12	23.3
S4-7	0.0764	21,704	13.5	22.9
Fitting results of *RSF*	*d* = 0.1491; *e* = −0.1738; *f* = 0.2018; *g* = 0.1269; *R*^2^ = 0.6574			

**Table 7:** The predicted and measured cumulative rut depth results of the different load units of test sections S1–S4.

Load Unit ID	Predicted Rut Depth (mm)	Measured Rut Depth (mm)	Prediction Error (mm)
S1-1	14.55	10.35	4.20
S1-2-1	16.58	15.4	1.18
S2-1	14.07	11.52	2.55
S2-2-1	17.59	19.3	−1.71
S3-1	7.98	8.77	−0.79
S3-2	12.90	13.92	−1.02
S3-3	17.42	16.28	1.14
S3-4	16.85	16.4	0.45
S3-5	17.02	16.71	0.31
S3-6	17.41	17.9	−0.49
S3-7	18.62	18.01	0.61
S4-1	9.53	10.4	−0.87
S4-2	15.33	19.23	−3.90
S4-3	23.38	25.54	−2.16
S4-4	25.89	25.83	0.06
S4-5	26.21	26.72	−0.52
S4-6	27.16	27.08	0.07
S4-7	27.60	27.47	0.13

## Data Availability

Data is presented in the article.
